# Cerebral astroblastoma with oligodendroglial-like cells: A case report

**DOI:** 10.1097/MD.0000000000027570

**Published:** 2021-10-29

**Authors:** Jian Gu, Yihua Wang, Juanhan Yu

**Affiliations:** a Department of Pathology, the First Affiliated Hospital of China Medical University,Shenyang, China.; b Department of Pathology, College of Basic Medical Sciences, China Medical University, Shenyang, China.

**Keywords:** astroblastoma, case report, oligodendroglial-like cells

## Abstract

**Rationale:**

Astroblastoma is a rare tumor of the central nervous system with uncertain biological behavior and origin. Its histopathological features have been well established, while, to our knowledge, astroblastoma with oligodendroglial-like cells have not been reported.

**Patient concerns:**

A 15-year-old girl presented with nausea, vomiting, headache, and visual disturbance.

**Diagnosis:**

Magnetic resonance imaging revealed a large neoplasm in the left temporal. Histologically, the tumor showed solid and pseudopapillary structure. Immunohistochemical staining showed that the tumor cells were positive for glial fibrillary acidic protein and vimentin. The oligodendroglial-like cells were positive for glial fibrillary acidic protein, vimentin, and oligodendrocyte transcription factor 2. The antigen KI67 labeling index was about 4%. Sequencing for isocitrate dehydrogenase (IDH) 1 codon 132 and IDH2 codon 172 gene mutations showed negative results. Furthermore, fluorescent analysis revealed neither 1p nor 19q deletion in the lesion. Based on these findings, the girl was finally diagnosed as astroblastoma.

**Interventions:**

A craniotomy with total excision of the tumor was performed.

**Outcomes:**

The follow-up time was 1 year, no evidence of disease recurrence was found in magnetic resonance imaging.

**Lessons:**

Cerebral astroblastoma with oligodendroglial-like cells is a clinically rare tumor of central nervous system. Clear distinction and diagnosis are critical.

## 1 Introduction

Today, according to the 2016 edition of the World Health Organization Classification of Tumors of the Central Nervous System,^[1]^ astroblastoma is belonging to the “other glioma” category. It can occur in persons of any age, but developing in children and young adults.^[2,3]^ Astroblastoma usually develops in cerebral hemispheres, but also in other parts of nervous system.^[4]^ On imaging examination, astroblastomas are well-demarcated masses.^[5]^ Although the microscopic description of astroblastoma in the existing literature is not completely consistent, there are 2 points that can be unified: the perivascular pseudorosette of tumor cells with short and stout cytoplasmic processes, radiating towards central blood vessels that often demonstrate sclerosis; and glial fibrillary acidic protein (GFAP)-positive expression.^[6,7]^ Herein, we report an extremely rare case of astroblastoma accompanied by oligodendroglial-like cells.

## 2 Materials and methods

The resected specimens were fixed with 10% neutral-buffered formalin and embedded in paraffin blocks. Tissue blocks were cut into 4 μm slides, deparaffinized in xylene, rehydrated with graded alcohols, and immunostained with the following antibodies: cytokeratin, GFAP, mutant isocitrate dehydrogenase (IDH)1 R132H, soluble protein-100, vimentin, synaptophysin (Syn), oligodendrocyte transcription factor 2 (Olig-2), alpha-thalassemia/mental retardation syndrome X, neuronal nuclear antigen (NeuN), tumor protein 53 (P53), and antigen KI67 (MaiXin, China). Then, the sections of each specimen were stained with streptavidin–peroxidase (KIT-9720, Ultrasensitive TM S-P, MaiXin, China) following the manufacturer's directions. The chromogen used was diaminobenzidine tetrahydrochloride substrate (DAB kit, MaiXin, China). All samples were slightly counterstained with hematoxylin, dehydrated, and mounted. For the negative controls, each sample was incubated with PBS instead of the primary antibody, as described above.

We performed fluorescent in situ hybridization (FISH) to check for deletions of chromosomes 1p and 19q. Dual color-probe hybridization was performed with Vysis 1p36/1q25 and 19q13/19p13 FISH Probe Kit (Abbott Molecular, IL) according to the manufacturer's instructions. At least 100 nonoverlapping nuclei were counted; samples were considered to be 1p- or 19q-deleted when >30% of counted nuclei presented 1 target (red) signal and 2 reference (green) signals. Sanger sequencing was used to detect the mutation of IDH 1 and 2 genes.

## 3 Case report

**Figure 1:**
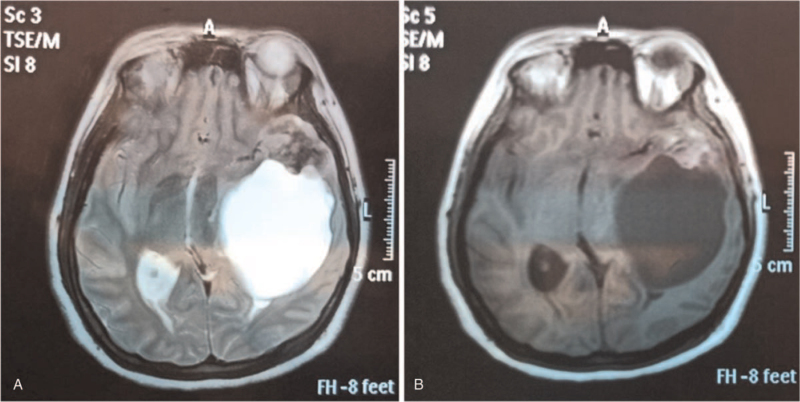
MRI showing a 7.5 × 5.0 × 5.0 cm sized cystic-solid mass in the in the left temporal. Cystic part of the tumor with a uniform long T1 and long T2 signalsss and the edge of is smooth. The solid part of the tumor with not regular edge shows inhomogeneous signal which is slight hypointense on T1-weigted images and slight hyperintense on T2-weighted images. There is no edema signal around the tumor. Tumor occupying effect is obvious, the left ventricle and lateral fissure pool are narrowly compressed and partially invisible. (Tumor occupying effect is obvious, compression of left ventricles and lateral fissure cistern narrowing or disappearance.). The midline structure shift to the right. There was no abnormality in the inner table of the adjacent skull. (A: Axial T1-weighted image. B: Axial T2-weighted image.) MRI = magnetic resonance imaging.

A 15-year-old Chinese female presented with nausea, vomiting, and vertigo for 1 year. Recently, these symptoms gradually aggravate with headache and hypopsia for 1 month. All laboratory test results were normal. Magnetic resonance imaging revealed a large, well-circumscribed, 7.5 × 5.0 × 5.0 cm size cystic-solid lesion in the left temporal. The tumor appeared hyperintense on T1- and T2-weighted images. The signal of most cystic parts of the tumor is uniform, the solid part is uneven. The tumor compression left ventricles and lateral fissure cistern, the midline structure moves to the right (Fig. 1). Radiological diagnosis was “other astrocytic tumor, pilocytic astrocytoma or pleomorphic xanthoastrocytoma”. A craniotomy with total excision of the tumor was performed. The follow-up time was 1 year, no evidence of disease recurrence was found in magnetic resonance imaging.

**Figure 2:**
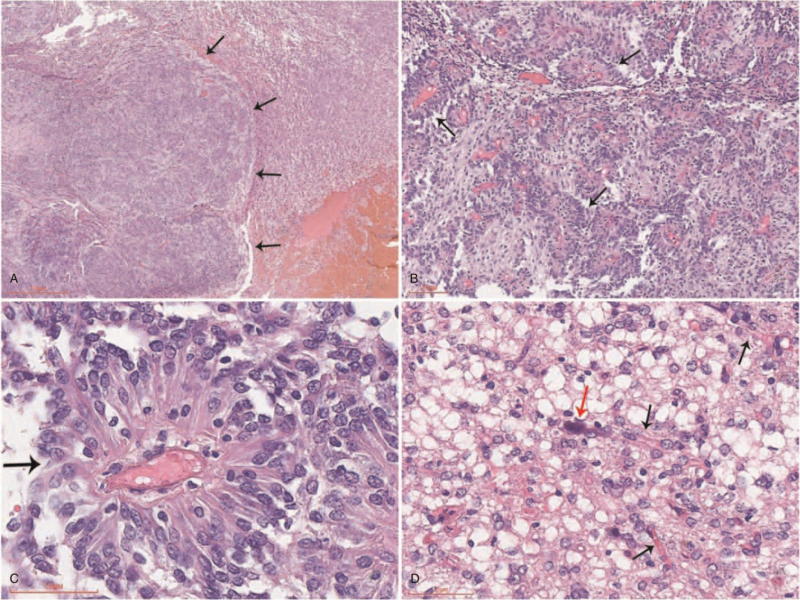
Histopathological findings. (A) Border of astroblastoma with oligodendroglial-like cells area was well defined. (B) The tumor was composed of poorly cohesive tumor cells forming solid or pseudopapillary structure. (C) Astroblastoma area: stout processes extend to the central vessels, forming astroblastic pseudorosettes. (D) Oligodendroglial-like cells area: the tumour cells showed a clear perinuclear halo with delicate “chicken-wire” network of branching capillaries (black arrow) and microcalcification (red arrow).

Small grayish-red fragments of the resected lesion were sent for histological examination. In histological examination, the lesion had 2 patterns in different proportions, astroblastoma area, and oligodendroglial-like cells area, a clear boundary between them (Fig. 2A). In the astroblastoma area, the tumor was composed of poorly cohesive tumor cells forming solid or pseudopapillary structure (Fig. 2B). Importantly, elongated tumor cells having broad footplates were characterized clustered around blood vessels, forming astroblastic pseudorosettes. These cells often seemed polarized, with the nucleus on 1 end and a tail-like cytoplasmic process on the other, possessed abundant eosinophilic cytoplasm and mitosis was rare (Fig. 2C). The tumor tissue was no necrosis or calcification. Blood vessels with no endothelial cell hyperplasia or hyalinization change. Significantly, oligodendroglial-like cells with clear cytoplasm, perinuclear halos, and round nuclei are observed, formed oligodendroglial-like honeycomb appearance, and the mitosis rate is usually low (Fig. 2D).

**Figure 3:**
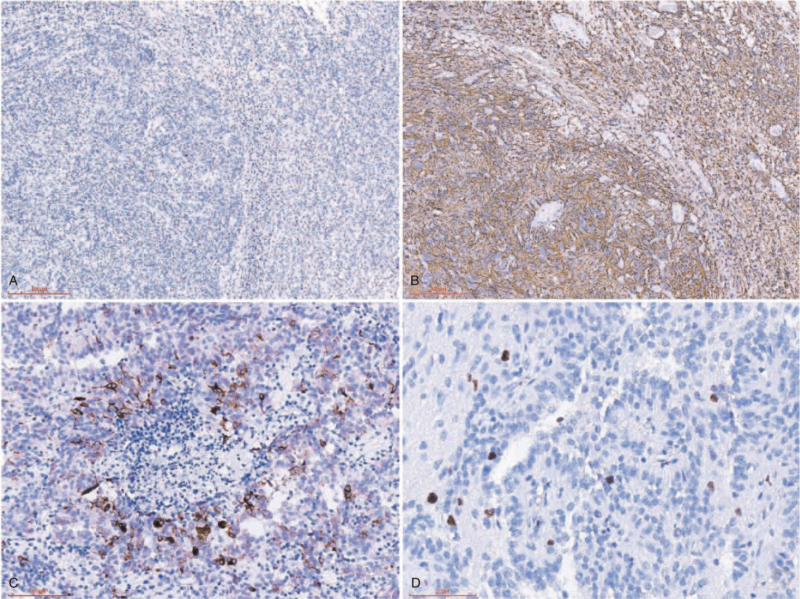
Immunohistochemistry findings. (A) The tumor cells were negative for IDH1 R132H. (B) The tumor cells and the peripheral oligodendroglial-like cells were positive for GFAP. (C) The tumor cells were membranous staining for EMA. (D) The Ki-67 proliferation index was about 4%. GFAP = glial fibrillary acidic protein, KI-67 = antigen KI67.

Immunohistochemical staining showed that the astroblastoma cells were negative for IDH1 R132H (Fig. 3A), cytokeratin, NeuN, Syn, P53, and Olig-2. However tumor cells were revealed positivity for vimentin, soluble protein-100, and alpha-thalassemia/mental retardation syndrome X, GFAP strong positive in the cytoplasm (Fig. 3B). Some tumor cells was positive for epithelial membrane antigen (EMA) in cell membrane (Fig. 3C). The oligodendroglial-like cells were positive for Olig-2, negative for IDH R132H, GFAP, EMA, NeuN, P53, and Syn. The antigen KI67 proliferation index was about 4%. There were no IDH1/2 mutations in the present tumor. FISH analysis revealed in this lesion with no 1pand 19q deletion. Based on these findings, the patient was diagnosed with astroblastoma with oligodendroglial-like cells.

## 4 Discussion

Since the original of astroblastoma was reported by Bailey and Cushing, there has been a lot of controversy about its existence and origin.^[8]^ Based on microscope observation, astroblastoma was classified as a transitional type between the astrocytoma and the glioblastoma multiform initially.^[9]^ Some scientists believed that GFAP and vimentin positivity in astroblastomas supported the hypothesis that this tumor was derived from the cytogenetically more primitive astroblast,^[10,11]^ or arises from a process of dedifferentiation involving mature astroglial cells.^[12]^ However, an ependymal or tanycyte derivation of astroblastomas understandably is considered by many authors observed the electron microscopic features.^[13,14]^ The tanycyte has been suggested as glial precursor cells and may occur during normal human embryogenesis, which explains the existence of congenital astroblastoma.^[15,16]^ As astroblastoma is often mixed with other types of tumor cells, such as glioblastomas or anaplastic astrocytomas, and the pseudo-chrysanthemum cluster structure also appears in other tumors, there has been controversy about its existence.^[17]^

But as the technology of gene identification matures, more and more specific genes are identified in astroblastoma. The most frequent genes alterations detected were meningioma 1 mutation, gains of chromosome arm 20q, and chromosome 19, losses on 9q, 10, and X.^[18–20]^ These suggested that astroblastomas represent a distinct entity with characteristic cytogenetic features that differ from those of ependymomas and astrocytomas. Because astroblastomas do not have gene detection for large sample cases, there is no unified gene mutation spectrum. It also shows tumors with histologic features of astroblastoma may result from diverse and possibly distinct genetic events.^[21]^

However, many of tumors present with perivascular pseudorosettes and can be confused with each other, such as ependymomas and papillary meningiomas. In our case, astroblastomas exhibiting broad footplates as opposed to the tapering processes seen in ependymoma. In contrast also to ependymomas, the spaces between the pseudorosettes were often rarified. In previous literature, EMA expression especially localized at membrane in astroblastomas, which is same with our case.^[22–24]^ In ependymoma, EMA express along the luminal surface of some ependymal rosettes or manifesting as dot-like perinuclear or ring-like cytoplasmic structures.^[25]^ Therefore, we ruled out the diagnosis of ependymoma by morphology and immunohistochemistry. The distinction between astroblastomas and papillary meningiomas is aided by immunohistochemical features that astroblastomas show positive staining with GFAP.^[26]^ Interestingly, in our case, there are oligodendrocyte-like areas outside the papillary areas, and the boundaries are clear. While, we found neither IDH 1/2 mutation nor 1p/19q codeletion, so we ruled out the diagnosis of oligodendroglioma. Based on these findings, we diagnosed this tumor as astroblastoma with oligodendroglial-like cells.

Oligodendroglial-like cells can appear in many central nervous system tumors, such as dysembryoplastic neuroepithelial tumor,^[27]^ rosette-forming glioneuronal tumor,^[28]^ papillary glioneuronal tumor,^[29]^ diffuse leptomeningeal glioneuronal tumor,^[30]^ gangliogliomas.^[31]^ Especially, some of these tumors such as gangliogliomas and dysembryoplastic neuroepithelial tumors represent the most frequent epileptogenic tumors in children and young adults.^[32]^ Unlike these, our patients did not have epilepsy. In the previous literature, only Lehman et al^[33]^ reported that oligodendroglial-like cells appeared in astroblastoma, but it appeared as oligodendrocyte-like cells appeared between astrocytoma cells, which is different from our case.

In the previous literature, different terminology has been used to describe oligodendroglial abnormalities including oligodendroglial hyperplasia,^[34]^ clusters of oligodendroglia,^[35]^ oligodendroglial hamartoma,^[36]^ and oligodendroglial-like cells. These lesions may represent a spectrum of the same abnormality. These lesions may represent a spectrum of the same abnormality. But the performance of oligodendroglial-like cells under the microscope is not the same, visible oligodendroglial-like cells floated in the mucus-like matrix, or infiltrated in the tumor tissue, or arranged in bundles. Oligodendroglial-like cells and oligodendroglioma have different gene mutation spectrum. Despite oligodendroglial-like morphology, it does not necessarily have chromosome 1p and 19q deletion, lacking the characteristic of IDH1 mutation in oligodendroglioma.^[37]^ Therefore, scientists believe that there may be different production mechanisms. In our case, there was no IDH1/2 mutation, or 1p and 19q deletion. And there was a clear boundary between the tumor tissue and oligodendroglial-like cells. We believe that the oligodendroglial-like cells here are not oligodendroglioma components, but oligodendroglial-like cells hyperplasia.

Previous case reports showed us that astroblastoma is aggressive tumor with a tendency to recur locally after surgical resection. They have suggested that adjuvant chemotherapy and radiotherapy can improve the survival rate.^[38]^ To our knowledge, astroblastoma with oligodendroglial-like cells is reported for the first in the literature, and the prognosis of the patient needs further follow-up.

## 5 Conclusions

In summary, we described a rare case of astroblastoma which accompany with oligodendroglial-like cells areas. Therefore, expanding the scope of pathological examination is necessary for correct diagnosis. In the process of diagnosis, we emphasize that the diagnosis of rare astroblastoma cannot be ignored because of common oligodendroglial-like cells.

## Author contributions

**Conceptualization:** Juanhan Yu.

**Methodology:** Yihua Wang.

**Writing – original draft:** Jian Gu.

**Writing – review & editing:** Juanhan Yu.
